# Obesity Affects the Biopsy-Mediated Detection of Prostate Cancer, Particularly High-Grade Prostate Cancer: A Dose-Response Meta-Analysis of 29,464 Patients

**DOI:** 10.1371/journal.pone.0106677

**Published:** 2014-09-03

**Authors:** Meng-Bo Hu, Sheng-Hua Liu, Hao-Wen Jiang, Pei-De Bai, Qiang Ding

**Affiliations:** 1 Department of Urology, Huashan Hospital, Fudan University, Shanghai, China; 2 Urology Research Institute, Fudan University, Shanghai, China; Innsbruck Medical University, Austria

## Abstract

**Background and Objectives:**

In previous studies**,** obesity (measured according to the body mass index) has correlated inconsistently with the risk of biopsy-measured prostate cancer, and specifically high-grade prostate cancer. This meta-analysis aimed to clarify these correlations.

**Methods:**

A comprehensive literature search of the MEDLINE and EMBASE databases was conducted for relevant studies published through January 2014. The pooled estimates of odds ratios (OR) and confidence intervals (CI) were computed, and the meta-analysis was performed with the STATA software according to a random effects approach.

**Results:**

A total of 11 studies that included 29,464 individuals were identified. A 5-kg/m^2^ increase in body mass index was associated with a 15% (OR, 1.15; 95% CI, 0.98–1.34) higher risk of prostate cancer detection and a 37% (OR, 1.37; 95% CI, 1.19–1.57) higher risk of high-grade prostate cancer detection at biopsy. There were no differences among the results of studies conducted in the USA, Europe or Asia. We also found that studies that had adjusted for prostate-specific antigen levels, digital rectal examination results, and prostate volumes obtained positive significant outcomes (OR, 1.27; 95% CI, 1.12–1.44), whereas studies that did not adjust for the above-mentioned confounding variables obtained negative results (OR, 0.92; 95% CI, 0.68–1.25). Moreover, the positive correlation between body mass index and the detection of both prostate cancer and high-grade diseases tended to be stronger as the number of biopsy cores increased.

**Conclusion:**

The present meta-analysis demonstrated that a high body mass index correlated positively with prostate cancer detection, especially high-grade prostate cancer detection. The adoption of a modified and possibly more aggressive biopsy strategy was suggested for obese populations.

## Introduction

Obesity, an increasing public health concern worldwide, has been linked to the development of various cancers [1]. This link has been further strengthened by fundamental research studies, in which scientists found that obesity could result in the generation of a unique endocrine and biochemical tumor growth-promoting microenvironment [2]. According to recent global estimates, prostate cancer (PCa) accounts for slightly more than a quarter of a million deaths annually [3]. Therefore, the relationship between PCa and obesity has attracted considerable attention from urologists. However, until now, several controversies concerning this relationship have persisted. Some previous studies have revealed a significant association between obesity and a higher incidence of PCa [4], a worse pathologic outcome [5], a greater risk of biochemical recurrence after treatment [6], [7] and a higher incidence of cancer-specific mortality [1]. In contrast, other studies have not shown similar correlations between obesity and PCa [8].

Might obesity be associated with the risk of PCa or high-grade PCa (HGPCa; Gleason score ≥7) diagnosis from a prostate biopsy? The results that might address this question have been inconsistent; different studies have found positive, null, or negative impacts of obesity on the risks of PCa and HGPCa. These contradictory findings have introduced some concern that the current screening practices might be less effective for detecting PCa among men with high body mass indices (BMI), thus suggesting that obese patients would be more likely to receive delayed diagnoses and experience worse pathological outcomes and prognoses.

To address this issue, we performed a dose-response meta-analysis of BMI and the risk of PCa and HGPCa at biopsy. The significance of this analysis could be extensive, as an investigation of the mechanisms behind these risk factors might help to optimize the current PCa screening strategies, particularly those for populations of overweight and obese men.

## Methods

### Search Strategy

We searched the MEDLINE and EMBASE databases for studies published up until January 10^th^, 2014. Our search criteria included English-language studies that addressed the relationship between obesity and the risk of PCa in a biopsied population. The search query was as follows: (“obese” OR “obesity” OR “overweight” OR “body mass index” OR “weight”) AND (“prostate”) AND (“biopsy”). References from retrieved reviews, meta-analyses and other relevant publications were also sought for inclusion in this study.

### Study Selection

Two investigators (Hu and Liu) independently assessed the eligibility of each study. The primary goal of our study was to confirm the existence of a positive correlation between obesity and the risk of PCa or HGPCa in a biopsied population. Therefore, only clinical studies conducted in analytical epidemiological settings that offered direct comparisons between the BMI and the risk of PCa or HGPCa were incorporated into the analysis. First, we screened the studies to remove duplicates and then excluded non-English-language articles, non-human studies, studies of other diseases, reviews, editorials, meta-analyses and case reports. Next, we again reviewed the databases and excluded studies that were not related to our topic, that had not been performed in an analytical epidemiology setting or that had not considered the BMI and biopsy results as the primary parameters. Subsequently, we performed a full-text review and excluded studies of all-cancer populations, those that focused on repeat prostate biopsies and those that failed to provide odds ratios (OR), relative risks (RR) or 95% confidence intervals (CI). In cases of overlap among the populations of different studies, only the most recent and most comprehensive data were included.

### Data Extraction and Quality Assessment

The following data were extracted from each study: title, authors, journal, publication year, study design, study period, study country, study population and setting, biopsy indications, biopsy methods and cores, BMI categories, numbers of subjects and cancer cases, OR estimates with corresponding 95% CIs and confounders that had been adjusted in the multivariate analyses. Two investigators (Hu and Liu) independently retrieved the data, and disagreements between them were resolved by consensus. If multiple multivariate logistic regression models were used in a study, the model with the highest number of variables was selected. To evaluate the study quality, we adopted the Newcastle–Ottawa Scale (NOS) with a nine-star system; this scale assesses the quality of cohort and case-control studies.

### Statistical Analysis

The dose-response meta-analysis was performed according to the method proposed by Orsini [9]; this method accounts for correlations between the log OR estimates across BMI categories. Each BMI category was assigned a value representing the median provided by the original research study. For studies that did not contain median values, we used the midpoints for closed categories and the same amplitudes as neighboring categories for open-ended categories. For studies in which the BMI values were only divided into two open-ended categories, we assumed that the OR and CI estimates for the higher BMI category were similar to those for a 5-kg/m^2^ increase in the BMI. We combined the ORs for each 5-kg/m^2^ increase in BMI according to the random-effect model. We assessed the inter-study heterogeneity using the Q and I^2^ statistics [10], wherein I^2^ values of 25%, 50% and 75% corresponded to the cut-off points for low, moderate and high degrees of heterogeneity, respectively. A subgroup meta-analysis was performed and stratified according to the study design, study location, number of biopsy cores and adjustment of the key confounders. A sensitivity analysis was conducted by omitting one study at a time and repeating the analysis to generate an estimate for comparison with the original results. Publication bias was evaluated using both Begg’s and Egger’s tests [11].

All analyses were performed using the STATA/SE 12.0 software (StataCorp, College Station, TX, USA). Statistical significance was defined as a two-tailed alpha value <0.05.

## Results

### Literature Search, Study Characteristics and Quality Assessment

**Figure 1:**
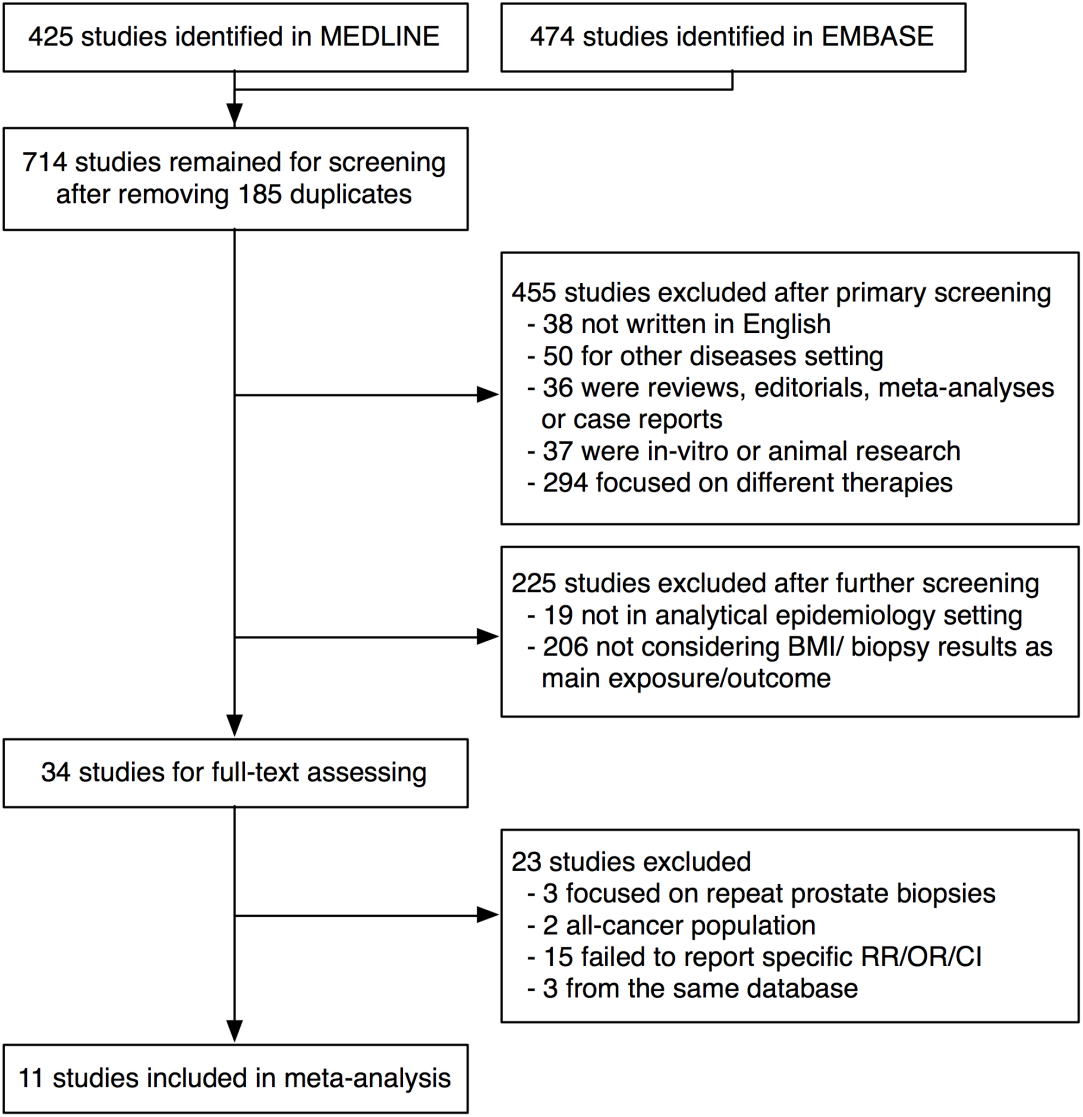
Flowchart showing the selection of studies for the meta-analysis.

According to the search query, 425 and 474 studies were identified from the MEDLINE and EMBASE databases, respectively. After excluding 185 duplicates, our literature search yielded a total of 714 studies that were potentially relevant to our topic. The detailed selection process is presented in a flowchart (Fig. 1). We excluded 455 and 225 studies after the primary and secondary screenings, respectively. Next, we obtained 34 studies with full-text assessments and excluded 23 of these based on the study goals and eligibility criteria. Eventually, 11 studies [12]–[22] were selected for the meta-analysis. Their baseline characteristics are listed in Table 1. These 11 studies, which were published from 2005–2013, included a total of 29,464 prostate biopsy patients and were conducted as prospective cohort studies (n = 2), retrospective cohort studies (n = 6) and case-control studies (n = 3). These studies were conducted in the United States (n = 4), Korea (n = 3), Italy (n = 2) and Japan (n = 2). The biopsy indications mainly comprised elevated prostate-specific antigen (PSA) and abnormal digital rectal examination (DRE) findings, whereas only one study considered hypoechoic lesions visualized using transrectal ultrasonography (TRUS). The numbers of biopsy cores ranged from six to ten when the prostate biopsies were performed before 2004; since 2004, all studies seem to have adopted the extended biopsy (biopsy cores ≥12) as a standard procedure. The studies were adjusted for different potential confounders, including age, PSA levels, DRE findings, and prostate volumes (PV), among others. The NOS scores ranged from eight to nine with a median score of nine.

**Table 1:** Baseline characteristics and quality assessment of included studies.

Author/year[reference]	Studydesign	Country	Studyperiod	Biopsyindication	Biopsy(cores)	No. ofsubjects/PCa/HGPCa	OR per5 kg/m^2^ increaseof BMI (95%CI) in PCa	OR per5 kg/m^2^increaseof BMI(95% CI) in HGPCa	Main confoundersadjusted^a^	NOS
**Park/2013** [12]	Cohort^c^	Korea	2008–2013	PSA≥4 ng/ml orpositive DRE	12	1213/344/203	1.45 (1.05–1.99)	1.50 (1.02–2.20)	1, 2, 3, 4	8
**De Nunzio/2013** [13]	Cohort^b^	Italy	2008–2011	PSA≥4 ng/ml orpositive DRE	12	668/257/118	1.28 (1.00–1.61)	1.69 (1.22–2.29)	1, 2, 3, 4, 13	9
**Oh/2013** [14]	Cohort^c^	Korea	2004–2011	PSA≥3 ng/mL, abnormalDRE or hypoechoiclesions in TRUS	≥12	3471/1102/538	1.30 (1.02–1.65)	NA	1, 2, 3, 4, 8	8
**Masuda/2013** [15]	Cohort^c^	Japan	2001–2011	PSA = 4–20 ng/ml orpositive DRE	≥14	3966/1689/561	1.37 (1.19–1.57)	1.43 (1.17–1.76)	1, 2, 3, 4, 6, 7, 9	8
**Jeon/2012** [16]	Cohort^c^	Korea	2003–2011	PSA≥4 ng/ml orpositive DRE	12	354/90/63	1.28 (0.72–2.27)	2.91 (0.88–9.60)	1, 2, 4, 12	8
**De Nunzio/2011** [17]	Cohort^b^	Italy	2005–2011	PSA≥4 ng/ml orpositive DRE	12	885/363/209	0.83 (0.59–1.10)	1.59 (1.15–2.20)	1, 2, 3, 4, 10	9
**Fowke/2011** [18]	Case-control	USA	NA	NA	NA	1203/NA/332	NA	1.12 (1.01–1.25)	1, 4, 5, 6, 7	8
**Freedland/2011** [19]	Case-control	USA	NA	NA	NA	6524/NA/NA	NA	1.28 (1.01–1.63)	1, 2, 3, 4, 5, 11	8
**Freedland/2008** [20]	Cohort^c^	USA	1999–2003	Elevated PSA orabnormal DRE	≥6	441/NA/NA	1.34 (1.05–1.69)	1.28 (0.90–1.84)	1, 2, 3, 4, 5, 13	8
**Gong/2006** [21]	Case-control	USA	1993-2003	PSA>4 ng/ml, abnormalDRE or end-of-study biopsy	≥6	10258/1936/521	0.99 (0.90–1.08)	NA	1, 5, 6, 11, 12	8
**Kobayashi/2005** [22]	Cohort^c^	Japan	2000-2004	Elevated PSA orabnormal DRE	≥10	481/208/NA	0.65 (0.44–0.96)	NA	1, 2, 4	8

*PCa* prostate cancer, *HGPCa* high-grade prostate cancer (Gleason≥7), *OR* odds ratio, *BMI* body mass index, *CI* confidence interval, *NOS* Newcastle-Ottawa Scale, *PSA* prostate specific antigen, *DRE* digital rectal examination, *TRUS* transrectal ultrasound, *NA* not available.

a Main confounders adjusted for: 1. age 2. PSA 3. DRE 4. PV 5. race 6. family history 7. number of biopsy cores 8. hypoechoic lesion in TRUS 9. %fPSA 10. testosterone 11. treatment 12. comorbidities 13. study center;

b prospective cohort;

C retrospective cohort.

### Overall Analysis

**Figure 2:**
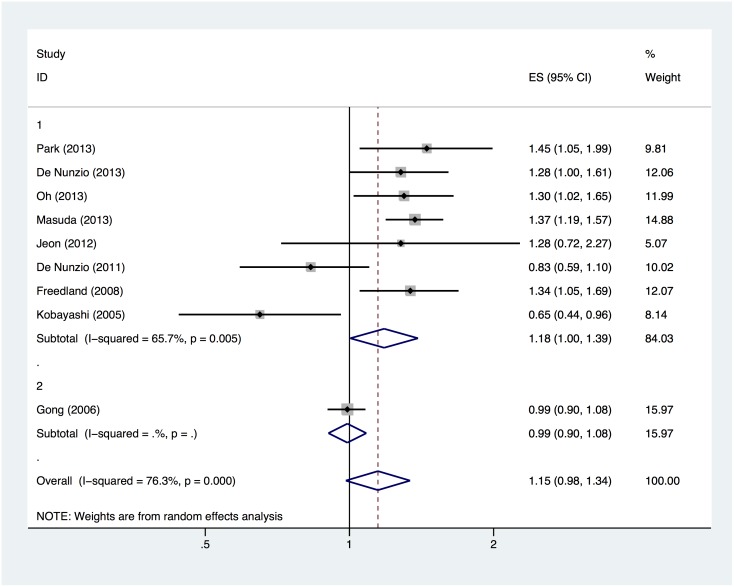
Forest plot of the odds ratio per 5-kg/m^2^ increase in the BMI and the risk of PCa among biopsied patients. Study ID: 1 = cohort study, 2 = case-control study.

**Figure 3:**
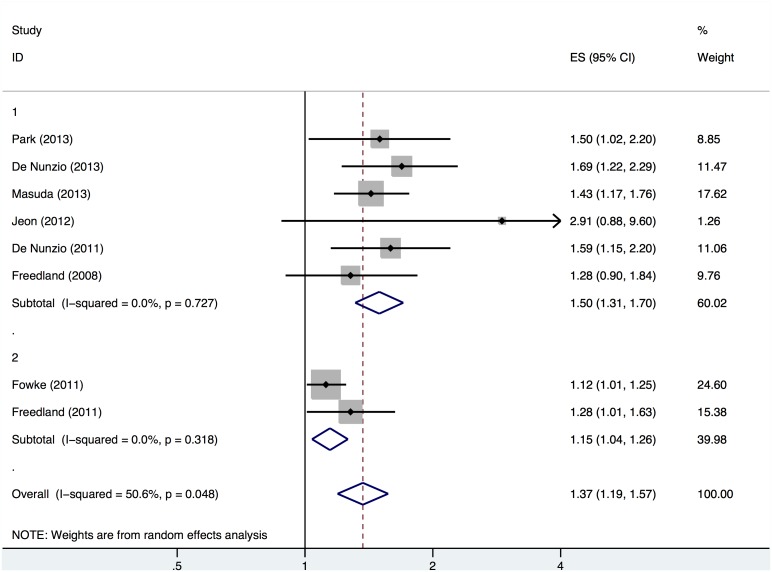
Forest plot of the odds ratio per 5-kg/m^2^ increase in the BMI and the risk of HGPCa in biopsied patients. Study ID: 1 = cohort study, 2 = case-control study.

Nine and eight studies were included in our meta-analyses when we modeled the relationships between the BMI and the risks of PCa and HGPCa, respectively. The results indicated that a 5-kg/m^2^ increase in the BMI was associated with a 15% higher risk of PCa (OR, 1.15; 95% CI, 0.98–1.34) among biopsied patients (Fig. 2), and high statistical heterogeneity was observed among the studies (Q = 33.77, P<0.001, I^2^ = 76.3%). Obesity appeared to have an even greater impact on the risk of HGPCa (OR, 1.37; 95% CI, 1.19–1.57; Fig. 3), although the statistical heterogeneity was modest (Q = 14.18, P = 0.048, I^2^ = 50.6%).

### Subgroup Analysis

The subgroup meta-analysis is presented in Table 2. When stratified by study design, the results revealed that the cohort studies yielded pooled ORs of 1.18 (95% CI, 1.00–1.39) and 1.50 (95% CI, 1.31–1.70) per 5-kg/m^2^ increase in BMI when correlated with the risks of PCa and HGPCa, respectively. The case-control studies yielded comparatively lower pooled ORs of 0.99 (95% CI, 0.90–1.08) and 1.15 (95% CI, 1.04–1.26). When stratified by study location, the correlations between the BMI and the risk of PCa were less clear in studies conducted in the USA (OR, 1.13; 95% CI, 0.84–1.51), Europe (OR, 1.04; 95% CI, 0.68–1.59) and Asia (OR, 1.20; 95% CI, 0.95–1.51). In contrast, all studies demonstrated a positive correlation between the BMI and the risk of HGPCa, regardless whether the study was conducted in the USA (OR, 1.15; 95% CI, 1.05–1.27), Europe (OR, 1.64; 95% CI, 1.31–2.06) or Asia (OR, 1.47; 95% CI, 1.23–1.75). We also found that the number of biopsy cores affected the correlations between obesity and the risks of PCa and HGPCa. When the total number of biopsy cores was ≥12, apparent positive correlations were observed between the BMI and the risks of PCa (OR, 1.25; 95% CI, 1.09–1.45) and HGPCa (OR, 1.53; 95% CI, 1.33–1.76). In contrast, the same positive correlations were comparatively weak when smaller numbers of biopsy cores were acquired. Meanwhile, we found that studies in which the PSA levels, DRE findings and PV had been adjusted obtained significantly positive outcomes (OR, 1.27; 95% CI, 1.12–1.44), whereas studies that did not adjust for the above-mentioned key confounders obtained negative outcomes (OR, 0.92; 95% CI, 0.68–1.25).

**Table 2:** Summary of ORs per 5-kg/m^2^ increase in the BMI and the risk of PCa and HGPCa among biopsied patients in subgroup analysis.

Subgroup	PCa					HGPCa				
	No. of studies	Pooled OR (95% CI)^a^	Heterogeneity test			No. of studies	Pooled OR (95% CI)^a^	Heterogeneity test		
			Q	P	I^2^ (%)			Q	P	I^2^ (%)
All studies	9	1.15 (0.98–1.34)	33.77	<0.001	76.3	8	1.37 (1.19–1.57)	14.18	0.048	50.6
*Study design*										
Cohort	8	1.18 (1.00–1.39)	20.42	0.005	65.7	6	1.50 (1.31–1.70)	2.82	0.727	0.0
Case-control	1	0.99 (0.90–1.08)	-	-	-	2	1.15 (1.04–1.26)	1.00	0.318	0.0
*Study location*										
USA	2	1.13 (0.84–1.51)	5.42	0.020	81.6	3	1.15 (1.05–1.27)	1.35	0.510	0.0
Europe	2	1.04 (0.68–1.59)	4.69	0.030	78.7	2	1.64 (1.31–2.06)	0.07	0.791	0.0
Asia	5	1.20 (0.95–1.51)	13.10	0.011	69.5	3	1.47 (1.23–1.75)	1.34	0.513	0.0
*Biopsy cores (all≥12)*										
Yes	6	1.25 (1.09–1.45)	8.94	0.111	44.1	5	1.53 (1.33–1.76)	1.98	0.739	0.0
No or NA	3	0.99 (0.74–1.33)	10.51	0.005	81.0	3	1.15 (1.05–1.27)	1.35	0.510	0.0
*Adjusted for all key confounders (PSA, DRE, PV)*										
Yes	6	1.27 (1.12–1.44)	9.04	0.108	44.7	6	1.43 (1.28–1.61)	2.74	0.740	0.0
No	3	0.92 (0.68–1.25)	5.12	0.077	60.9	2	1.49 (0.63–3.50)	2.43	0.119	58.9

*PCa* prostate cancer, *HGPCa* high-grade prostate cancer (Gleason≥7), *OR* odds ratio, *NA* not available, *PSA* prostate specific antigen, *DRE* digital rectal examination, *PV* prostate volume.

a Pooled odds ratio was calculated using random effects model.

### Dose-Response Analysis

**Figure 4:**
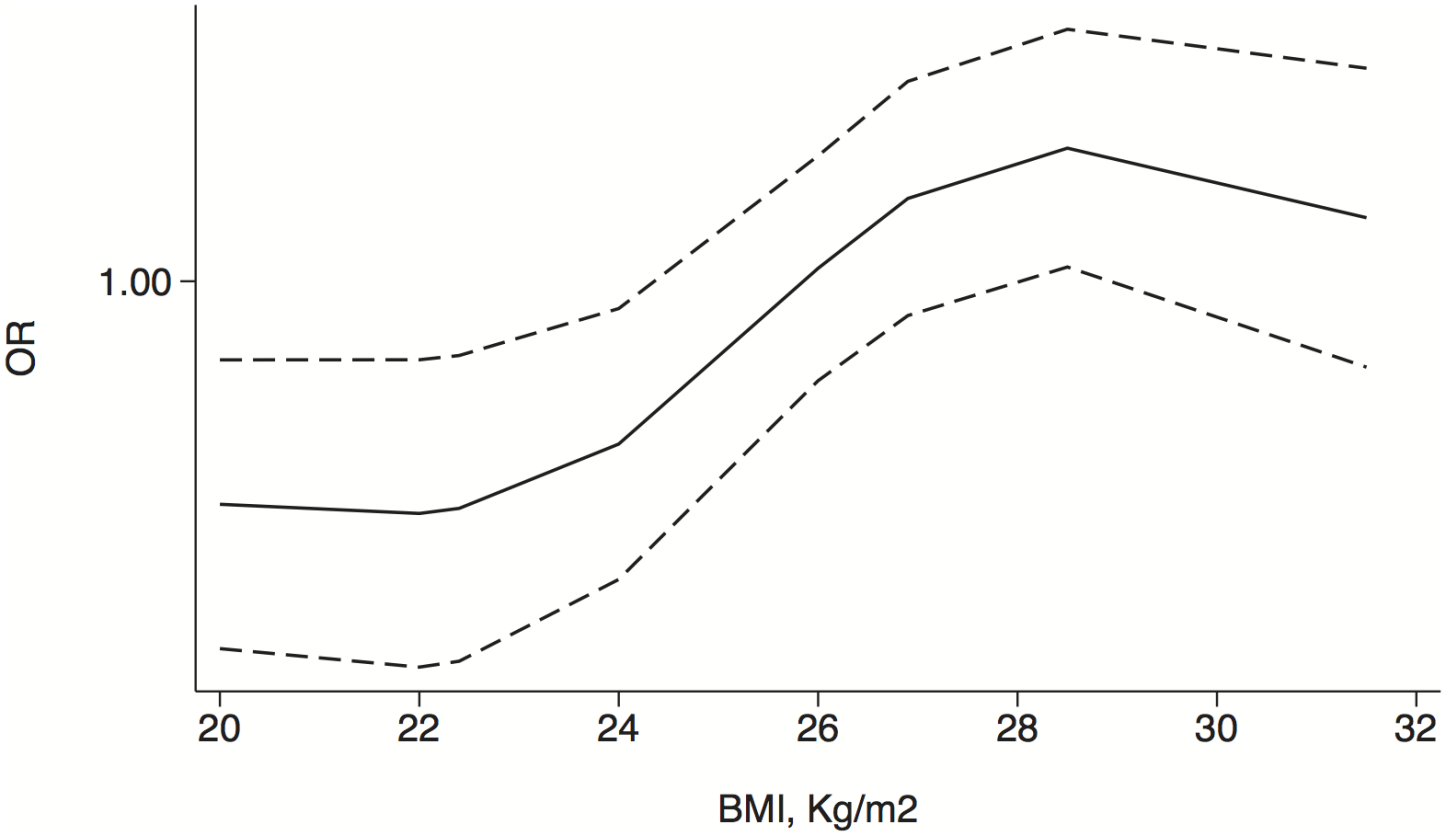
Dose-response relationship between the BMI and risk of PCa in biopsied patients. The adjusted odds ratio (solid line) and 95% confidence interval (dashed lines) are indicated.

**Figure 5:**
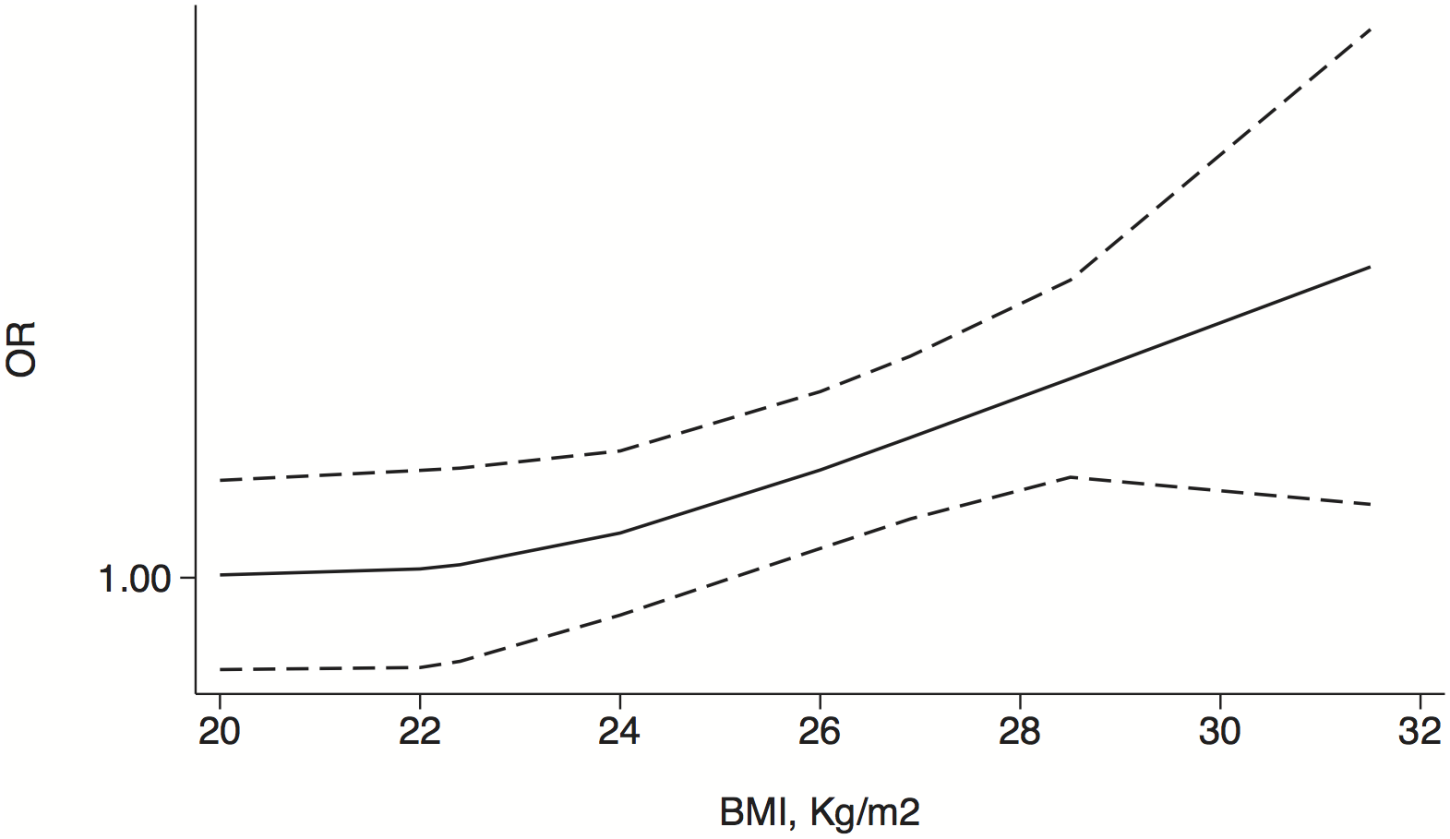
Dose-response relationship between the BMI and risk of HGPCa in biopsied patients. The adjusted odds ratio (solid line) and 95% confidence interval (dashed lines) are indicated.

Studies that provided BMI categories and ORs were pooled in a random-effect dose-response meta-analysis, thus revealing the various associations between the BMI and the risks of PCa and HGPCa. The correlation between the BMI and the risk of PCa appeared to be slightly complex (Fig. 4). Specifically, a steady and increasing trend was observed for BMI values <29. However, that trend was reversed for BMI values >29. With regard to the risk of HGPCa, the slope remained nearly consistent, and a near-linear relationship between the BMI and the risk of HGPCa was demonstrated (Fig. 5).

### Sensitivity and Publication Bias Analyses

Given the high level of heterogeneity (I^2^ = 76.3%) observed in the meta-analysis of the relationship between BMI and the risk of PCa, we performed a sensitivity analysis by excluding each study in turn. Upon excluding the study with the highest weight (15.97%) [21] from the random-effect meta-analysis, the resulting pooled OR was 1.18 (95% CI 1.00–1.39); therefore, this exclusion did not significantly change the result. We evaluated the publication bias using Begg’s test (p = 0.602) and Egger’s test (p = 0.681) and found no evidence of such bias. Moderate heterogeneity (I^2^ = 50.6) was identified with respect to the correlation between the BMI and the risk of HGPCa; however, a sensitivity analysis indicated that this heterogeneity was not due to the incorporation of a single study. Furthermore, the contradictory outcomes obtained with Begg’s test (p = 0.536) and Egger’s test (p = 0.006) indicated probable publication bias.

## Discussion

To our knowledge, the present study was the first meta-analysis of the relationship between obesity and the risk of PCa and HGPCa diagnosis from biopsy. This analysis summarized the results of 11 clinical studies, eight of which were published within the last three years. As the outcomes of previous studies have been inconsistent, the general analysis demonstrated the tendency toward a positive although statistically insignificant correlation between the BMI and the risk of PCa (OR, 1.15; 95% CI, 0.98–1.34), and a positive, statistically significant correlation between the BMI and the risk of HGPCa (OR, 1.37; 95% CI, 1.19–1.57). Our analysis, which included a total of 29,464 patients, featured great power for evaluating these relationships. The introduction of various subgroup analyses and dose-response relationships in our study is also noteworthy.

With respect to the locations of the studies that analyzed the relationship between obesity and the risk of PCa, only previous reports from developed countries such as the USA, in which the populations were heavier, consumed higher-fat diets and performed less exercise, were likely to indicate a positive correlation (if at all). However, the current subgroup meta-analysis did not find any differences in the pooled results among the studies conducted in different locations. Consequently, we observed only a tendency towards a positive correlation between the BMI and the risk of PCa, regardless whether the studies were conducted in the USA, Europe or Asia; however, we did observe a positive and statistically significant correlation between the BMI and the risk of HGPCa. Masuda [15] attributed the equal PCa risks among Asians and Westerners to the fact that Asians tend to carry a higher percentage of body fat at a given BMI and therefore have a greater propensity towards central obesity [23], a factor that was proven to be even more closely associated with increased risks of both PCa and HGPCa. Moreover, the limited number of studies restricted us from reaching any additional conclusions regarding the issue of study location.

Furthermore, we hypothesized that the positive correlation between the BMI and PCa might be attributable to both biological and technical issues. Various biological mechanisms are involved in the PCa initiation and progression. Obese patients produce smaller amounts of total testicular testosterone, a finding that has a complex relationship with PCa and has been associated with a poorer pathological outcome [24]. Moreover, the adipokines and inflammatory cytokines secreted by adipose tissue have been shown to enhance tumor growth [25]. Additionally, changes in the endocrine and metabolic microenvironments of obese patients lead to compensatory hyperinsulinemia and increased levels of bioavailable insulin-like growth factor 1, both of which have been linked to the promotion of carcinogenesis and the inhibition of apoptosis [26]. The results of our study agreed with these reports; although we failed to identify a statistical correlation between the BMI and PCa, the increasing trend towards a positive correlation between the BMI and HGPCa might be explained by these biological factors.

Because the correlation between the BMI and PCa was less evident than the correlation between the BMI and HGPCa, we further hypothesized that this difference might be due to technical issues associated with PCa detection. Early PCa detection depends on prostate biopsy, which is indicated by elevated PSA levels (commonly ≥4 ng/ml) or abnormal DRE findings. However, statistics have demonstrated that obese patients have lower PSA levels and larger prostates; additionally, it is more difficult to observe abnormal DRE findings in obese patients because of the presence of perirectal fat. Under such circumstances, patients with high BMI values who underwent biopsies based on typical indications or for whom the standard numbers of biopsy cores were collected would very likely experience delayed diagnoses and poor prognoses. The current subgroup analysis supported our hypothesis. After adjusting the pooled results of the studies for all key confounders (PSA, DRE and PV), we observed a strong shift in the OR relative to the studies that omitted one or two of the key confounders (OR 1.27; 95% CI, 1.12–1.44 vs. OR, 0.92; 95% CI, 0.68–1.25); this finding indicates that these confounders obscured PCa detection to some extent.

An inverse relationship between the BMI and PSA levels has been widely recognized [27], [28]; this relationship may be due to lower testosterone levels [29] or a hemodilution effect resulting from the larger plasma volumes in overweight and obese individuals. Bañez [30] reported that the PSA concentration decreased significantly with an increasing BMI whereas the estimated total PSA mass did not, suggesting that the lower PSA concentrations in overweight and obese men might be explained by larger plasma volumes. The hemodilution theory was further validated in the baseline data collected from 28,380 men enrolled in the Prostate, Lung, Colorectal, and Ovarian (PLCO) Cancer Screening Trial [31]. In that study, the PSA concentrations decreased significantly with increasing BMI (P<0.001); however, the total PSA mass showed no association with BMI (P = 0.10). Therefore, if obese men were subjected to the same indications for prostate biopsy, their low PSA levels would reduce the sensitivity of PCa screening and might result in reduced rates of PSA-driven biopsy as well as delayed diagnoses and poor pathological outcomes [32], [33]. However, the actual PSA levels in these obese patients might have been higher than the levels measured at biopsy. This possibility should be taken into account when considering the hemodilution theory and might also explain the increasing risk of PCa, particularly HGPCa, in the biopsied population.

Meanwhile, a positive correlation between the BMI and PV has also been reported [29]. A high PV was found to reduce the rate of PCa detection [34], as it was more difficult to detect cancer via biopsy in a larger prostate. In a multicenter series of patients who had been treated via radical prostatectomy, a larger prostate size was estimated to cause detection failure in up to 25% of all PCa cases [29]. Importantly, this bias might be overcome simply by obtaining more biopsy cores from obese individuals or by using a scale to determine the number of biopsy cores according to the prostate size [35]. Our results showed that the pooled ORs increased with a higher number of biopsy cores (OR, 1.25; 95% CI, 1.09–1.45 vs. OR, 0.99; 95% CI, 0.74–1.33). This result could be explained by the fact that patients with smaller prostates might derive greater benefit from a higher number of biopsy cores or by the different baseline characteristics.

The current meta-analysis suggested that not only were obese men underdiagnosed but also that this population presented with more aggressive tumors at diagnosis. From our perspective, technical detection bias and biological causes contribute jointly to the elevated risks of PCa and HGPCa in obese patients. Therefore, the maintenance of a healthy weight and the development of novel medications that target obesity-related molecules or signaling pathways should be encouraged. More importantly, we should consider the use of modified biopsy indications and improved biopsy methods for obese patients. Given that patients with higher body weights exhibited lower PSA levels, it could therefore be suggested that for obese men, a lower PSA threshold (≤4 ng/ml) should be used when discriminating between malignant and benign diseases of the prostate. Some studies have proposed formulas for calculating the relationship between obesity and PSA levels [36], [37]; however, more studies are needed to validate these formulas. As the prostate size tends to increase in obese patients, it might be necessary to adopt more aggressive biopsy strategies such as the collection of more biopsy cores or the integration of magnetic resonance imaging-guided biopsy.

The strength of the present meta-analysis was attributed to the strict eligibility criteria, the application of the NOS, the large numbers of subjects and cases, and the assessment of potential nonlinear relationships. Nonetheless, several limitations should be considered when interpreting our data. First, our literature search and data extraction were restricted to studies published in indexed journals, and the number of included studies restricted us from formulating additional conclusions in the subgroup analysis. Second, the present analysis exhibited moderate to severe heterogeneity that might be explained by the various confounding factors present in the original papers, including the different study designs, baseline characteristics, biopsy indications and numbers of biopsy cores. Third, given the lack of data, the current study could not evaluate measures of abdominal obesity such as the waist circumference and waist-to-hip ratio, although these measures are considered to be more strongly associated than BMI with hormonal and metabolic alterations [38], [39]. Additionally, the association between obesity duration and PCa detection was not assessed.

In conclusion, our analysis suggests a positive correlation between BMI and PCa detection, particularly HGPCa detection, at biopsy. Biopsied patients with higher BMI values had a greater risk of receiving diagnoses of PCa and HGPCa. The maintenance of an ideal body weight might help to improve the rate of early detection and the prognoses of individuals with PCa. More importantly, the insight into the biopsy-associated technical issues associated, including the lower PSA levels and larger prostate size associated with a higher BMI, indicated a potential risk for delayed diagnosis and poor pathological outcomes in obese patients following treatment according to standard biopsy strategies. Therefore, we highly suggest the adoption of modified and possibly more aggressive biopsy indications and methods for men with higher BMI values (e.g., decreasing the PSA threshold for biopsy indication or collecting more biopsy cores). Additional large population studies across multiple institutions and countries will be needed to obtain a unified picture of the factors responsible for this observed increase. Finally, additional randomized clinical trials should focus on exploring modified prostate biopsy indications and methods based on BMI categories.

## Supporting Information

Checklist S1 **PRISMA Checklist.**
(DOC)
